# T-2 Toxin/HT-2 Toxin and Ochratoxin A ELISAs Development and In-House Validation in Food in Accordance with Commission Regulation (EU) No 519/2014

**DOI:** 10.3390/toxins9120388

**Published:** 2017-11-30

**Authors:** Michalina Oplatowska-Stachowiak, Tim Kleintjens, Nermin Sajic, Willem Haasnoot, Katrina Campbell, Christopher T. Elliott, Martin Salden

**Affiliations:** 1 EuroProxima B.V., Arnhem 6827 BN, The Netherlands; tim.kleintjens@europroxima.com (T.K.); nermin.sajic@europroxima.com (N.S.); m.salden@noviosmart.eu (M.S.); 2 RIKILT Wageningen UR, Wageningen 6708 WB, The Netherlands; willem.haasnoot@wur.nl; 3 Institute for Global Food Security, School of Biological Sciences, Queen’s University Belfast, Belfast BT9 5BN, UK; katrina.campbell@qub.ac.uk (K.C.); chris.elliott@qub.ac.uk (C.T.E.)

**Keywords:** mycotoxins, enzyme-linked immunosorbent assay, immunoassay, screening

## Abstract

T-2 toxin/HT-2 toxin (T-2/HT-2) and ochratoxin A (OTA) are mycotoxins that can contaminate a variety of agricultural commodities. To protect consumers’ health, indicative limits for T-2/HT-2 and maximum limits for OTA have been set by the European Commission, requiring food business operators and controlling agencies to conduct routine checks for the presence of these harmful contaminants. Screening methods are increasingly used for monitoring purposes. Due to the demand for new and improved screening tools, two individual detection methods, T-2/HT-2 and OTA enzyme-linked immunosorbent assays (ELISAs), were developed in this study. The T-2/HT-2 ELISA was based on a T-2 monoclonal antibody with an IC_50_ (50% inhibitory concentration) of 0.28 ng/mL and 125% cross-reactivity with HT-2. As regards the OTA ELISA, a new sensitive monoclonal antibody specific to OTA with an IC_50_ of 0.13 ng/mL was produced. Both developed ELISA tests were then validated in agricultural commodities in accordance with the new performance criteria guidelines for the validation of screening methods for mycotoxins included in Commission Regulation (EU) No 519/2014. The T-2/HT-2 ELISA was demonstrated to be suitable for the detection of T-2/HT-2 in cereals and baby food at and above the screening target concentration (STC) of 12.5 μg/kg and 7.5 μg/kg, respectively. The OTA ELISA was shown to be applicable for the detection of OTA in cereals, coffee, cocoa and wine at and above the STC of 2 μg/kg, 2.5 μg/kg, 2.5 μg/kg and 0.4 ng/mL, respectively. The accuracy of both ELISAs was further confirmed by analysing proficiency test and reference samples. The developed methods can be used for sensitive and high-throughput screening for the presence of T-2/HT-2 and OTA in agricultural commodities.

## 1. Introduction

Mycotoxins are toxic secondary metabolites of fungal origin which have a major effect on agriculture and ecology, as well as human and animal health [1,2]. The main mycotoxin producing fungi belong to *Aspergillus*, *Fusarium* and *Penicillium* species and they can contaminate different agricultural crops and commodities in the field, but also during storage, processing and transport. Mycotoxins can then enter the feed and food chain resulting in chronic and acute exposure of humans and animals to these toxic contaminants. To protect human health regulatory limits have been set for the most harmful mycotoxins in many countries worldwide. In the European Union, there are maximum, indicative or guidance limits for aflatoxins, ochratoxin A (OTA), fumonisins, zearalenone, deoxynivalenol, T-2 toxin/HT-2 toxin (T-2/HT-2), citrinin and patulin in food and feed commodities [3,4,5].

T-2/HT-2 are among the most potent of the trichothecenes type of mycotoxins produced by *Fusarium* species which can affect a variety of cereal grains [6]. T-2 is rapidly metabolised to HT-2 in vivo by animals, plants and fungi and both toxins commonly co-occur in agricultural commodities [7]. Due to their high stability T-2 and HT-2 are also present in the processed products of the cereal grains. These toxins are harmful to both humans and animals, among which cats and pigs are the most sensitive species. T-2 and HT-2 impair protein and DNA synthesis and interfere with both red and white blood cell formation in bone marrow leading to a number of adverse effects. Based on these toxic effects the Panel on Contaminants in the Food Chain (CONTAM Panel) of the European Food Safety Authority set the tolerable daily intake for the sum of both T-2 and HT-2 at 100 ng/kg body weight [7]. In the European Union, in accordance with Commission Recommendation 2013/165/EU [4], Member States, with the involvement of food and feed business operators are required to monitor for the presence of both T-2 and HT-2 in food and feed. The indicative limits for T-2 and HT-2 were also established based on the occurrence data (Table 1). If the sample is found to be contaminated above these values, it is required to perform an investigation in order to identify factors causing these high levels. According to Commission Recommendation 2013/165/EU [4], the limit of quantification of a method for T-2/HT-2 analysis should not be higher than 5 μg/kg for T-2 and HT-2 individually in processed cereals and 10 μg/kg in unprocessed cereals. The limit of detection of an analytical screening method should not be higher than 25 μg/kg for the sum of T-2 and HT-2. There are various analytical methods for the quantitative detection of T-2 and HT-2 such as high-performance liquid chromatography (HPLC) [8] or gas chromatography (GC) [9] coupled to a range of different detectors, and liquid chromatography–tandem mass spectrometry (LC-MS/MS) [10,11]. Immunochemical methods [12,13] such as ELISAs [14,15,16,17,18,19,20], membrane-based immunoassays [21,22] and surface plasmon resonance (SPR) biosensor [23] have also been developed. These tests do not require extensive sample preparation procedure and the large investment in equipment and can be used as high-throughput screening tools before confirmatory analysis.

Ochratoxin A (OTA) is a mycotoxin produced by a number of *Penicillium* and *Aspergillus* species that can contaminate different agricultural commodities prior to harvest and more often during storage [24]. OTA occurs in various cereals, pulses, coffee beans, spices, dried fruits, grape juice and wine. The International Agency for Research on Cancer classified OTA as possibly carcinogenic to humans (Group 2B) [25]. OTA is known to be nephrotoxic, immunotoxic, neurotoxic and teratogenic [24]. To protect consumers’ health in the European Union maximum levels (MLs) for OTA were set for different food commodities [5]. The MLs vary from 0.5 to 20 μg/kg depending on the food type (Table 2). The guidance limits for feed are 250 μg/kg for cereals and cereal products and 50 and 100 μg/kg for complete and complementary feedstuffs for pigs and poultry, respectively [3]. A number of methods have been developed for the detection of OTA in food and feed and they include HPLC [26,27] and LC-MS/MS [28]. Methods such as ELISAs [29,30,31,32,33,34,35,36,37] and membrane-based testes [33,38] have been also established for rapid screening.

ELISA methods remain the gold standard for food screening in many countries. End users such as food manufacturers and control agencies continue to use ELISA for screening purposes as this remains a cost-effective and high-throughput method. New and improved ELISA kits for mycotoxins are, however, continuously needed to fulfil the requirements of changing regulations. These changes can include anything from the adjustments of regulatory limits to updates in validation criteria, but also extending the list of food commodities that require testing. Therefore, the first aim of this study was to develop two new and improved ELISA test kits, the first one for the detection of T-2/HT-2 and the second one for screening for the presence of OTA in a range of agricultural commodities. For the development of a T-2/HT-2 ELISA method that complies with the related European regulations, cross-reactivity with both T-2 and HT-2 is required, as these toxins occur together and the indicative limits concern the sum of both of them. Therefore, the new direct competitive T-2/HT-2 ELISA established in this study was based on a monoclonal antibody [21,23] with good cross-reactivity with T-2 and HT-2 and with sufficient sensitivity to fulfil the requirement to detect at least 25 μg/kg [4]. With regards to the development of a new test for OTA, the important factors were assay sensitivity and low cross-reactivity with ochratoxin B (OTB), which is a less toxic analogue of OTA. Having a high cross-reactivity is undesirable in this case as it can lead to false positive results if both toxins are present in a sample. The maximum limits for OTA in food in the EU are very low, which then makes it necessary to use only extremely sensitive antibodies in ELISA tests. This is why a new, very sensitive monoclonal antibody was produced during this research. The OTA ELISA developed in this study was also intended to be applicable for the analysis of a range of food commodities: cereals, roasted, instant and green coffee, cocoa and wine without the need to use any time-consuming strategies such as solid phase extraction or immunoaffinity chromatography during sample preparation.

Apart from the development of new ELISA methods for the detection of T-2/HT-2 and OTA the final aim of this research was also to validate the developed assays in accordance with the new guidance for the validation of semi-quantitative screening methods included in Commission Regulation (EU) No 519/2014 [39]. Previously, the validation approach described in Commission Decision 2002/657/EC [40] and explained in Community Reference Laboratories (CRLs) Guidelines [41], has been used for many years for the validation of screening methods of analysis. However, Commission Regulation (EU) No 519/2014 incorporated new criteria for the validation of screening methods for mycotoxins. The European Commission recognized the increasing popularity of these methods in the field of mycotoxin analysis and addressed the need to establish new criteria for the performance of screening methods. Tests developed in this study meet these updated criteria and can be easily transformed into commercial assays.

## 2. Results and Discussion

### 2.1. T-2/HT-2 ELISA

#### T-2/HT-2 ELISA Sensitivity and Cross-Reactivity

**Figure 1:**
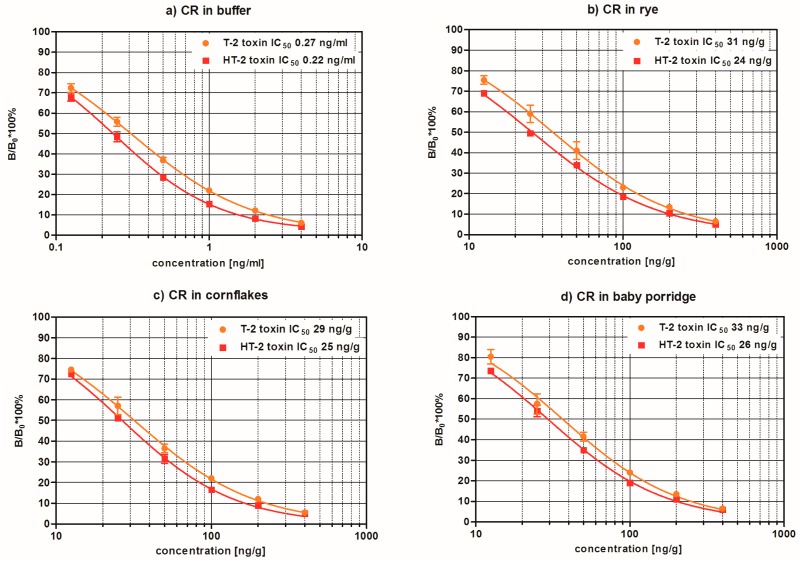
Cross-reactivity (CR) of the monoclonal antibody used in the T-2 toxin/HT-2 toxin ELISA in: (**a**) buffer; (**b**) extracted rye; (**c**) corn flakes; and (**d**) baby porridge matrices. B/B_0_, ratio of the absorbance corresponding to the given standard to the absorbance of the 0 ng/mL standard (maximum signal); IC_50_, 50% inhibitory concentration.

A monoclonal antibody specific to T-2/HT-2 was used to develop a direct competitive ELISA. A seven-point standard curve was prepared for T-2 at 0, 0.125, 0.25, 0.5, 1, 2 and 4 ng/mL (Figure 1a). The mean IC_50_ (50% inhibitory concentration, i.e., the concentration corresponding to 50% inhibition of the maximum signal, which is absorbance measured for 0 ng/mL standard) for T-2 was 0.28 ng/mL (*n* = 12). The linear range corresponding to IC_20_–IC_80_ was 0.09–1.14 ng/mL. IC_20_ and IC_80_ are the concentrations at 20% and 80% inhibition of the maximum signal, respectively. For most accurate analysis, samples showing more than 2 ng/mL should be further diluted and reanalysed to fit within the linear range.

Cross-reactivity with T-2 and HT-2 was determined in buffer and in matrix. Standard curves (*n* = 3) for T-2 and HT-2 in buffer and extracted standard curves from rye, corn flakes and baby porridge samples were assayed in parallel to investigate the influence of the matrix on the cross-reactivity profile (Figure 1). The cross-reactivity with HT-2 was calculated as the ratio of the IC_50_ of T-2 divided by IC_50_ of HT-2 multiplied by 100. The cross-reactivity with HT-2 was found to be 125% in buffer, 131% in rye, 117% in corn flakes and 128% in baby porridge. This cross-reactivity has to be taken into account when interpreting the results because, if the assay is based on a calibration using T-2 standards, the results of the analysis of unknown samples may be slightly overestimated if HT-2 is present. There was no cross-reactivity with other commonly occurring mycotoxins: aflatoxin B_1_, deoxynivalenol, fumonisin B_1_, ochratoxin A and zearalenone.

### 2.2. T-2/HT-2 ELISA Validation

**Figure 2:**
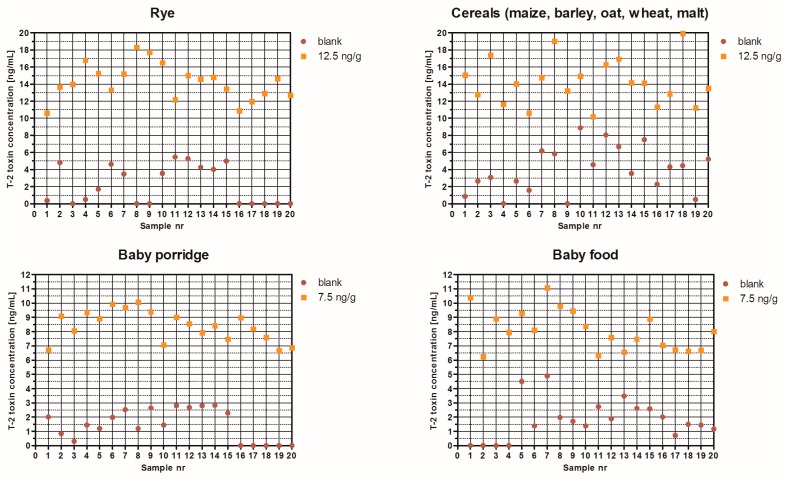
Validation of the T-2 toxin/HT-2 toxin ELISA in different matrices.

A mixture of methanol or acetonitrile in water has been used for the extraction of T-2 and HT-2 from cereals and cereal products [17,18,21]. In this study, 40% methanol in water was shown to extract toxins with good efficiency across a range of cereals. Further dilution in PBS buffer was used to reduce the matrix effects. The T-2/HT-2 ELISA was validated for each commodity type in accordance with the guidance for validation of semi-quantitative screening methods [39]. The summary results for the sets of blank samples and samples spiked at screening target concentration (STC) are presented in Table 3. There was no overlap observed between the blank and spiked samples sets for each matrix (Figure 2). The false suspected rates were typically low. Only the cereal group showed higher variation in results and as a consequence a false suspected rate of 2.5% at the set STC was observed. The T-2/HT-2 ELISA can be used to screen cereals and baby food for the presence of T-2/HT-2 at the level of 12.5 μg/kg and 7.5 μg/kg and above, respectively.

The results of the recovery and repeatability studies for the T-2/HT-2 ELISA are presented in Table 4. The recovery was determined in rye spiked at 12.5, 25 and 50 μg/kg with T-2 and baby porridge spiked at 7.5, 15 and 30 μg/kg. The mean recoveries were in the range of 99–114% and the coefficient of variation (CV) was lower than 20.2%, demonstrating good repeatability of the method. Commission Regulation (EU) No 519/2014 does not specify the performance criteria such as recovery and CV for semi-quantitative screening methods. For confirmatory methods, the recovery for T-2 and HT-2 at the concentration of 15–250 μg/kg should be between 60% and 130% and CV ≤ 30%. The developed T-2/HT-2 ELISA fulfils all these requirements.

**Figure 3:**
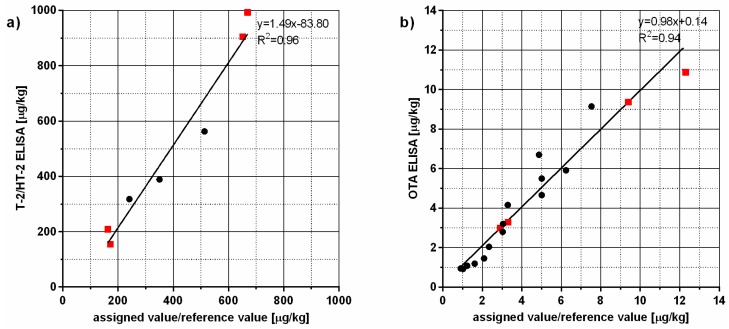
Correlation of the results obtained by: (**a**) T-2 toxin/HT-2 toxin (T-2/HT-2) ELISA; and (**b**) ochratoxin A (OTA) ELISA with assigned/reference values for proficiency testing (dots) and reference samples (squares).

Four proficiency testing samples: three oats and one rye; and two oats and two feed reference samples were analysed by the T-2/HT-2 ELISA and the results were compared to the assigned/reference values for these samples (Figure 3a). The correlation coefficient was calculated to be 0.96 and the slope of the regression line 1.49. All the analysed samples contained a mixture of T-2 and HT-2. Due to the 125% cross-reactivity of the antibody used in the ELISA with HT-2 one can expect the overestimation of the total T-2/HT-2 content by the T-2/HT-2 ELISA and that effect was demonstrated when analysing proficiency testing and reference samples. Nevertheless, the results obtained by the ELISA were well correlated with the assigned/reference values, indicating suitability of the developed assay as a screening method for the detection of T-2/HT-2 in different matrices.

In previous studies, ELISA methods were either developed only for specific T-2 detection with varying sensitivities [14,15,17,19,20] or they could detect both T-2 and HT-2 [16,18], but in a rather limited number of matrices. The most sensitive antibody specific to T-2 only with an IC_50_ of 0.12 ng/mL and 7% CR with HT-2 was a polyclonal produced by Wang et al. (2010) [17] and applied in ELISA for analysis of cereals and feed. Other T-2 specific antibodies were used to develop ELISAs for the detection of T-2 in feed, cereals and tissues [14]; rice [15]; cereals [19]; and barley [20]. These antibodies were characterised by an IC_50_ of 1.46, 22.09, 2.3 and approximately 100 ng/mL, respectively. As for the detection of both T-2 and HT-2 simultaneously, Yoshizawa et al. (2004) [18] developed an indirect competitive ELISA for analysis of wheat only using an antibody with an IC_50_ of 0.16 ng/mL. In another study Li et al. (2012) [16] developed a direct competitive ELISA for milk only based on a monoclonal antibody with an IC_50_ of 51.96 ng/mL. 

The new, direct competitive ELISA developed in this study is based on an antibody characterised by a very low IC_50_ of 0.28 ng/mL and it can be used to detect both T-2 and HT-2 simultaneously not only in cereals, but also in baby food with the sensitivity recommended by an EU regulation [4]. This ELISA is the first test for both T-2 and HT-2 validated in accordance with the new guidelines for the validation of semi-quantitative screening methods [39].

### 2.3. OTA ELISA

#### 2.3.1. Production of the Monoclonal Antibody to OTA

The spleens from three immunised mice were used in the fusion experiments. Approximately, 500 hybridomas were screened during each fusion. Four clones from the first and second fusion and three clones from the third fusion were found to have the highest degrees of sensitivity. After two rounds of cloning, these final cell lines were used to produce monoclonal antibodies. Each antibody stock at a concentration of 2 mg/mL was tested using a competitive antigen-coated ELISA. The standard curves for OTA were prepared in the range 0.001–1000 ng/mL. Antibody 9C12 showed the highest sensitivity and it was selected for the OTA ELISA development (Table 5).

#### 2.3.2. OTA ELISA Sensitivity and Cross-Reactivity

**Figure 4:**
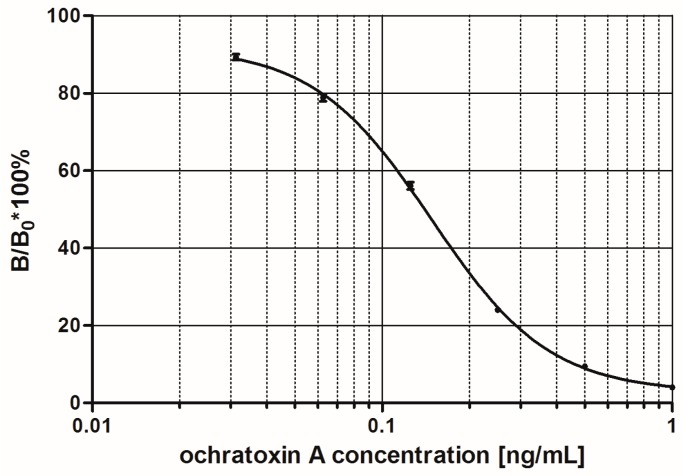
Typical standard curves for ochratoxin A in the ochratoxin A ELISA (*n* = 12). (B/B_0_, ratio of the absorbance corresponding to the given standard to the absorbance of the 0 ng/mL standard (maximum signal)).

The monoclonal antibodies to OTA were used to develop a direct competitive ELISA. A seven -point standard curve was prepared for OTA at 0, 0.0313, 0.0625, 0.125, 0.25, 0.5 and 1 ng/mL (Figure 4). The mean IC_50_ for OTA was 0.13 ng/mL (*n* = 12). The linear range corresponding to IC_20_–IC_80_ was calculated to be 0.06–0.29 ng/mL. To obtain the most accurate results samples showing more than 0.5 ng/mL needed to be diluted and reanalysed to fit within the linear range.

The cross-reactivity of the OTA monoclonal antibody was tested with ochratoxin B (OTB), a less toxic analogue of OTA and it was determined to be 18%. A high cross-reactivity is undesirable as it might lead to a higher rate of false suspected results. As the concentration of OTB is generally much lower than that of OTA [9], this 18% cross-reactivity should not lead to an increased rate of false suspected results. There was no cross-reactivity with the other commonly occurring mycotoxins: aflatoxin B_1_, deoxynivalenol, fumonisin B_1_, T2/HT2 and zearalenone.

#### 2.3.3. OTA ELISA Validation

OTA can contaminate different foodstuffs including cereals, coffee, cocoa and wine. Due to the differences in matrix composition between these food types, sample preparation methods were developed separately for each commodity type. For cereals, the best method was extraction with 50% methanol in PBS combined with defatting with n-hexane followed by dilution in assay buffer. Other matrices required extraction with acidified dichloromethane, followed by the extraction of OTA from dichloromethane to carbonate-bicarbonate buffer pH 9.6 and further dilution in assay buffer. 

**Figure 5:**
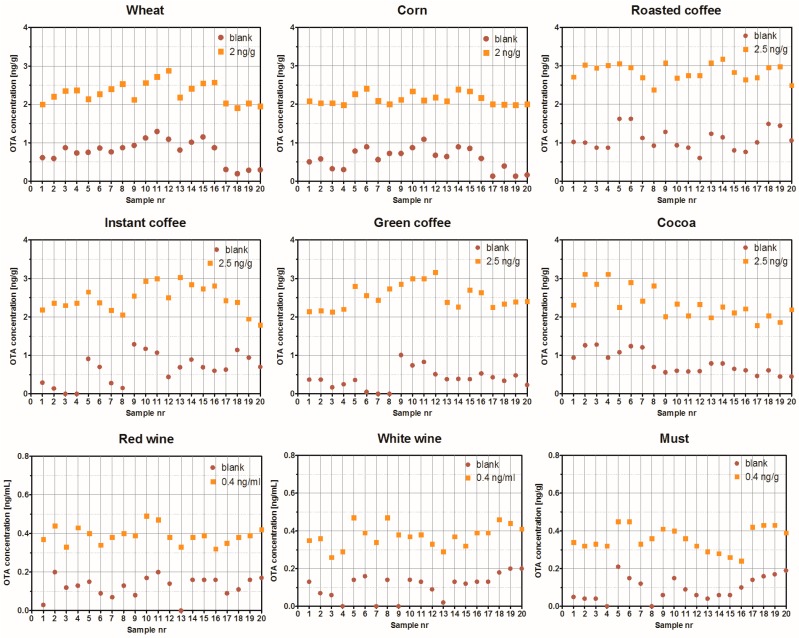
Validation of the ochratoxin A (OTA) ELISA in different matrices.

The OTA ELISA was validated for each commodity type in accordance with the guidance for validation of semi-quantitative screening methods [39]. The STCs were set to assure the most sensitive detection possible and all the chosen values were below the regulatory maximum limits set by Commission Regulation EC (No) 1881/2006 and amendments [5]. Twenty blank samples and 20 samples spiked at STC for each matrix were analysed. There was a complete separation between blank and spiked samples (Figure 5) and the calculated false suspected rates were below 1% (Table 6). This indicates the applicability of the OTA ELISA for the detection of OTA at and above set STCs in tested matrices with low number of blank or low contaminated samples falsely classified to contain OTA at the level higher than STC.

The results of the recovery and repeatability studies are presented in Table 7. The samples were spiked at different concentrations below, at and above the maximum limits set by Commission Regulation (EC) No 1881/2006 and amendments [5]. The recoveries found were in the range 68–115% and the CV lower than 17.2% demonstrating good repeatability. The OTA ELISA showed the ability to distinguish between different mycotoxins concentrations in nine different matrices below, at and above the maximum limits set in the EU [5]. The performance criteria: recovery and CV are not specified in Commission Regulation (EU) No 519/2014 for semi-quantitative screening methods. For confirmatory methods, the recovery for OTA at the concentration <1 μg/kg should be between 50% and 130% and CV ≤ 30%. For the concentrations at and above 1 μg/kg, the recovery should be between 70% and 110% and CV ≤ 20%. For the new OTA ELISA, the recovery was slightly above 110% only in the case of roasted coffee spiked at 2.5 and 5 μg/kg. The recovery was also slightly lower than 70% for must spiked at 1 μg/kg. The OTA ELISA was shown to fulfil the performance criteria for confirmatory methods for the rest of the matrices.

Fourteen FAPAS samples: five white wines, one rose, four roasted coffees, two green coffees and two maize samples with assigned values were analysed by the OTA ELISA. Additionally, two reference wheat samples and two reference maize samples were tested. The correlation coefficient was found to be 0.94 and the slope of the regression line close to 1, indicating excellent accuracy of the developed OTA ELISA for the analysis of a range of different matrices (Figure 3b).

A number of studies on the production of monoclonal and polyclonal antibodies specific to OTA and on the development of ELISA tests have been reported over recent years. The most sensitive monoclonal antibody to OTA was produced by Li et al. (2013) [30] displaying an IC_50_ of 0.058 ng/mL in an indirect competitive ELISA. The method was validated for the analysis of rice, wheat, soybean and peanut, however it required the application of expensive and time-consuming immunoaffinity chromatography columns during sample preparation in order to reduce matrix effects. Wang et al. (2007) [33] obtained a polyclonal antibody with an IC_50_ of 0.07 ng/mL and applied it in a direct competitive ELISA for the analysis of cereals, raisins, coffee beans, grape juice and beer. In other studies, antibodies with IC_50_ between 0.38 and 5 ng/mL were obtained and used to develop ELISA methods for the detection of OTA in corn and feed [29]; cereals, beans and coffee [35]; cereals [32]; dried figs [31]; chilli [36]; and green coffee [34].

The new OTA ELISA developed in this study was based on a monoclonal antibody with an IC_50_ of 0.13 ng/mL, which is one of the most sensitive antibodies reported in the scientific literature. The application of this antibody allowed for the development of a simple sample preparation methods for a wide range of commodities: cereals; roasted, instant and green coffee; cocoa; wine; and must. The extraction methods did not require application of any immunoaffinity chromatography or solid phase extraction for such complex matrices as roasted coffee and cocoa. This is the first OTA ELISA validated in accordance with the new guidelines for the validation of screening methods for mycotoxins [39]. This new OTA ELISA can be further validated for other agricultural commodities commonly affected by OTA contamination to make it even more versatile screening tool for the presence of OTA.

## 3. Conclusions

A monoclonal antibody against T-2 with an IC_50_ of 0.28 ng/mL and 125% cross-reaction with HT-2 was used to develop a direct competitive ELISA for the detection of both T-2 and HT-2 in cereals and baby food. A new sensitive monoclonal antibody against OTA with an IC_50_ of 0.13 ng/mL was produced in this study and applied in the development of a direct competitive ELISA for the detection of OTA in cereals, coffee, cocoa and wine. Sample preparation methods for OTA analysis in nine different matrices were developed and optimized without the requirement for any time-consuming sample purification strategies such as solid phase extraction or immunoaffinity chromatography. The required sensitivity was achieved using a liquid–liquid extraction approach and diluting the samples to reduce matrix effects. Both assays were validated in accordance with the new guidelines for validation of semi-quantitative screening methods for mycotoxins included in Commission Regulation (EU) No 519/2014. For the T-2/HT-2 ELISA, the STCs were set at 12.5 μg/kg for cereals and 7.5 μg/kg for baby food. The method was validated for the detection of T-2/HT-2 at a level of STC and above with low false suspected rate. The recoveries were also determined at different levels and they were between 99% and 114% with the CV lower than 20.2%. In the case of the OTA ELISA, the STCs were set at 2 μg/kg for cereals, 2.5 μg/kg for different types of coffee and cocoa and 0.4 ng/mL for wine and must. The method was shown to be suitable to detect OTA at STC and above with low false suspected rates. The recoveries for OTA were also tested at different levels and they were between 68% and 115% and the CV was lower than 17.2%. The good accuracy of both ELISAs was confirmed by analysing proficiency testing and reference samples. Both T-2/HT-2 ELISA and OTA ELISA were demonstrated to be fit for purpose, therefore these new methods can be applied for the sensitive and high-throughput screening for the presence of T-2/HT-2 and OTA in agricultural commodities.

## 4. Materials and Methods

### 4.1. Chemicals, Consumables and Apparatus

T-2 toxin, HT-2 toxin, ochratoxin A, ochratoxin B, aflatoxin B_1_, deoxynivalenol, fumonisin B_1_, zearalenone, methanol, hexane, dichloromethane, phosphoric acid, sulphuric acid, disodium hydrogen phosphate, sodium dihydrogen phosphate, potassium dihydrogen phosphate, sodium carbonate, sodium bicarbonate, sodium chloride, Tween 20, bovine serum albumin (BSA), keyhole limpet hemocyanin (KLH), horseradish peroxidase (HRP), gelatine, dicyclohexylcarbodiimide (DCC), *N*-hydroxysuccinimide (NHS) and dimethyl sulfoxide (DMSO) were purchased from Sigma-Aldrich (Dorset, UK and Zwijndrecht, The Netherlands). 3,3′,5,5′-Tetramethylbenzidine (TMB) was obtained from Neogen (Lansing, MI, USA).

A laboratory mill IKA A11 Basic was used for sample blending. Sigma 4K10 centrifuge was used for samples centrifugation and BioTek EL808 type ELISA plate reader for reading the microtiter plates.

### 4.2. Production of Toxin Conjugates

T-2 was conjugated to HRP in accordance with a method described in Li et al. (2012) [16].

OTA-BSA conjugate was obtained from Sigma (Dorset, UK). OTA-KLH and OTA-HRP were produced using the carbodiimide method. For OTA-KLH 0.8 mg (2 μmol) of OTA was dissolved in 300 μL of DMSO. Then 50 μL of 80 mM DCC/NHS solution in DMSO was added and the solution was mixed overnight at room temperature in the dark. The activated OTA was added slowly while mixing to 5 mg (1 nmol) of KLH dissolved in 1.7 mL of PB buffer pH 7.5 and allowed to react overnight at room temperature while being slowly mixed. The conjugate was finally dialysed against 0.9% sodium chloride for 3 days with 3 changes of dialysing buffer. The purified conjugate was stored at −20 °C. OTA-HRP was produced in a similar way by using 0.55 mg (1.362 μmol) of OTA and 10 mg (227 nmol) of HRP.

### 4.3. Monoclonal Antibodies

A T-2/HT-2 monoclonal antibody was produced and characterised in previous studies [21,23]. The antibody did not have any measurable cross-reactivity with T-2 triol, T-2 tetraol, deoxynivalenol, nivalenol, neosolaniol or diacetoxyscirpenol [23].

OTA monoclonal antibodies were produced in this study. Two mice per immunogen were immunised with OTA-BSA and OTA-KLH according to the previously described procedure [42]. The dose of the immunogen used was 15 μg per injection. Ten days after each immunisation the blood samples were collected from mice and screened in an antigen-coated assay [42] by using OTA-BSA or OTA-KLH at the concentration 1 μg/mL as a coating antigen to determine antibody titre and sensitivity. After the sufficient antibody titre was achieved fusion experiments were performed. Mice were euthanized and their spleens were removed and processed immediately. Three fusions were performed in total, one using spleen from mouse immunised with OTA-BSA and two from mice immunised with OTA-KLH. The fourth spleen was not used as the antibody titre from the immunised mice was low. The splenocytes were fused with SP2 myeloma cells using polyethylene glycol as a fusogen [43]. The exact fusion procedure and further screening, cloning, antibody production and purification procedures are described elsewhere [42]. The study received approval from the Queens University Belfast Animal Welfare and Ethical Review Body on 09/01/14 and the experiments included in the manuscript were conducted under project licence PPL2756 (date of issue: 12/02/14).

### 4.4. T-2/HT-2 ELISA

The microtiter strips of the plate were coated overnight with 10 μg/mL polyclonal rabbit anti-mouse antibody diluted in phosphate buffered saline (PBS pH 7.4). A seven-point standard curve for T-2 in assay buffer (PBS pH 7.4 with 1% BSA) was prepared in the range of 0–4 ng/mL. Standards or extracted samples (50 μL) were added in duplicate to the wells of the microtiter plate. A background control (100 μL of the assay buffer) was added to two wells. Then 25 μL of T-2-HRP (1 mg/mL stock diluted 1/40,000) and 25 μL of the antibody (1 mg/mL stock diluted 1/40,000) were added to each well apart from the background control wells. The plate was shaken for 30 s on a plate shaker and incubated at 37 °C in the dark for 1 h. Next the solution was discarded and the plate was washed 3 times with PBS containing 0.05% Tween 20. The colour was developed by adding 100 μL of TMB to each well and incubating the plate for 30 min at 20–25 °C in the dark. The enzymatic reaction was stopped with 100 μL of 0.5 M sulphuric acid added to each well. The plate was read at 450 nm on a microtiter plate reader.

### 4.5. OTA ELISA

The microtiter strips of the plate were coated overnight with 10 μg/mL polyclonal rabbit anti-mouse antibody diluted in phosphate buffered saline (PBS pH 7.4). A seven-point standard curve for OTA in the range 0–1 ng/mL in assay buffer (0.1 M PB buffer pH 8.0 with 1% gelatin) was prepared. Standards or extracted samples (50 μL) were added in duplicate to the wells of the microtiter plate. A background control (100 μL of the assay buffer) was added to two wells. Then, 25 μL the antibody (2 mg/mL diluted 1/40,000) and 25 μL of the OTA-HRP (2 mg/mL diluted 1/20,000) diluted in the assay buffer were added to each well apart from the background control wells. The plate was incubated at 20–25 °C in the dark for 1 h. Then, the solution was discarded and the plate was washed 3 times with PBS containing 0.05% Tween 20. The colour was developed by adding 100 μL of TMB to each well and incubating the plate for 30 min at 20–25 °C in the dark. The enzymatic reaction was stopped with 100 μL of 0.5 M sulphuric acid added to each well. The plate was read at 450 nm on a microtiter plate reader.

### 4.6. Samples

#### 4.6.1. Samples for T-2/HT-2 ELISA

One sample of each cereal: rye, maize, barley, oat, malt, wheat, baby porridge and four samples of baby cereals with different percentage of grain and milk content were purchased at local stores. The samples were confirmed to be free of T-2/HT-2 by LC-MS/MS (Trilogy Lab, Washington, MO, USA) [44].

For determination of the accuracy of the T-2/HT-2 ELISA the following 4 reference materials were used: oats containing 82 ± 4 μg/kg of T-2 and 81 ± 4 μg/kg of HT-2 was obtained from European Reference Materials (ERM); oats containing 85 ± 14 μg/kg of T-2 and 87 ± 12 μg/kg of HT-2, feed containing 265 ± 29 μg/kg of T-2 and 386 ± 68 μg/kg of HT-2, feed containing 273 ± 39 μg/kg of T-2 and 396 ± 50 μg/kg of HT-2 were obtained from Food Analysis Performance Assessment Scheme—FAPAS (Fera, Sand Hutton, UK). Additionally, 3 proficiency testing samples were also analysed: Oats with the assigned values of 137 μg/kg for T-2 and 104 μg/kg for HT-2 was obtained from FAPAS (Fera, Sand Hutton, UK); oats with the assigned values of 270 μg/kg for T-2 and 80 μg/kg for HT-2 and rye with the assigned values of 334 μg/kg for T-2 and 179 μg/kg for HT-2 were obtained from CODA-CERVA (Brussels, Belgium).

#### 4.6.2. Samples for OTA ELISA

Four samples of each commodity type: wheat, maize, roasted coffee, instant coffee, green coffee, white and red wine and cocoa powder were purchased at local stores. The samples were confirmed to be free of OTA by LC-MS/MS (Trilogy Lab, Washington, MO, USA) [44] or LC-FLD (Eurofins, Hamburg, Germany) [45]. For determination of the accuracy of the OTA ELISA, proficiency testing samples with assigned values were obtained from FAPAS (Sand Hutton, UK), and these were 5 white wine samples with the assigned values of 0.91, 1.02, 1.23, 1.61 and 2.34 μg/L; 1 rose sample with the assigned value of 2.09 μg/L; 3 roasted coffee samples with the assigned values of 3.29, 4.87 and 5.01 μg/kg; and 2 green coffee samples with the assigned values 6.24 and 7.53 μg/kg. Two wheat and 2 maize samples with reference values 3.3 ± 1.4 and 9.4 ± 3.0, and 2.9 ± 0.9 and 12.3 ± 3.0 μg/kg, respectively, were obtained from Trilogy (Washington, MO, USA).

### 4.7. Sample Preparation

#### 4.7.1. T-2/HT-2 ELISA Sample Preparation

*Cereals and baby food.* The extraction method was based on a procedure described in [23]. One gram of homogenised sample was weighed in a 15 mL polypropylene centrifuge tube. Five millilitres of 40% (*v*/*v*) methanol was added to each sample and the mixture was vortexed for 10 s. After centrifugation (10 min at 4000× *g*) 25 μL of supernatant was added to 475 μL of assay buffer (PBS pH 7.4 with 1% BSA) for cereals and 50 μL to 450 μL of assay buffer for baby food. The samples were then analysed by the T-2/HT-2 ELISA.

#### 4.7.2. OTA ELISA Sample Preparation

*Cereals.* Samples (100 to 200 g) of wheat and maize were homogenised in a laboratory blender. A 2 g sub-sample was weighed into a 50 mL polypropylene centrifuge tube. OTA was extracted with 8 mL of methanol:PBS buffer pH 7.4 (50:50, *v*/*v*) by mixing the sample for 15 min using head-over-head mixer. Two millilitres of n-hexane were also added to the sample during the extraction to remove fat. After centrifugation (5 min at 4000× *g*), the upper n-hexane layer was removed and 100 μL of the bottom layer was added to 400 μL of the assay buffer (PB buffer pH 8.0 with 1% gelatine). The sample was then centrifuged (5 min at 4000× *g*). The clear supernatant was used for analysis by the OTA ELISA.

*Roasted and instant coffee.* A 2 g homogenised sample was weighed into a propylene tube. A 10 mL aliquot of dichloromethane and 50 μL of 6 M phosphoric acid were added and the sample was mixed head-over-head for 15 min. Then the sample was centrifuged for 2 min at 4000× *g* and the supernatant was filtered through a filter paper. Two millilitres of the filtrate were collected into a 15 mL polypropylene tube and 1 mL of 0.05 M carbonate-bicarbonate buffer pH 9.6 was added. The sample was vortexed for 5 s and mixed head-over-head for 5 min. After centrifugation step (5 min, 4000× *g*) 50 μL of the upper layer was collected and added to 450 μL of the assay buffer (PB buffer pH 8.0 with 1% gelatine). Finally, the sample was centrifuged (5 min, 4000× *g*) and the supernatant was analysed by the OTA ELISA.

*Green coffee*. Two grams of homogenised sample were weighed into a propylene tube. A 10 mL aliquot of dichloromethane and 10 mL of 0.1 M phosphoric acid were added and the sample was mixed head-over-head for 15 min. The sample was centrifuged for 2 min at 4000× *g* and the bottom layer was filtered through a filter paper. Then, the procedure was the same as for roasted and instant coffee.

*Cocoa*. Two grams of homogenised sample were weighed into a propylene tube. A 10 mL aliquot of dichloromethane and 200 μL of 6 M phosphoric acid were added and the sample was mixed head-over-head for 15 min. The sample was centrifuged for 2 min at 4000× *g* and the supernatant was filtered through a filter paper. Then the procedure was the same as for roasted and instant coffee.

*Wine (red and white) and must.* One millilitre of wine or 1 g of must were pipetted into 15 mL polypropylene tube. Two millilitres of dichloromethane and 20 μL of 6 M phosphoric acid were added. The sample was vortexed for 5 s and mixed head-over-head for 15 min. The mixture was allowed to separate into two layers for 5 min. The upper layer was removed and 1 mL of the bottom dichloromethane layer was collected into a new tube and 0.5 mL of 0.05 M carbonate-bicarbonate buffer pH 9.6 was added. The sample was vortexed for 5 s and mixed head-over-head for 5 min. After centrifugation (5 min, 4000× *g*), 100 μL of the upper layer was collected and added to 300 μL of the assay buffer. The sample was then analysed by the OTA ELISA.

### 4.8. ELISAs Validation

In this study, the validation approach included in Commission Regulation (EU) No 519/2014 was followed [39]. This new Regulation is specifically intended for validation of semi-quantitative screening bioanalytical methods such as ELISA, lateral flow devices and immunosensors for the analysis of mycotoxins. The result of the measurement by these methods should be a numerical value. For these methods, the concept of STC is used. STC is a concentration of interest for detection of the mycotoxin. The Regulation requires to determine 2 parameters: a cut-off and a false suspected rate. The cut-off is the concentration measured in a sample, above which the sample is classified as “suspect”, meaning it may contain mycotoxin at a level higher than STC. The cut-off is calculated from the results obtained for 20 samples spiked at STC by using equation given in the Regulation [39]:

Cut-off = R_STC_ − t-value_0.05_ × SD_STC_

where R_STC_ is the mean response of the positive control samples at STC; t-value_0.05_ is the one tailed t-value for a rate of false negative results of 5%; and SD_STC_ is the standard deviation at STC.

The calculated cut-off allows for the 5% rate of false negative results, meaning that 1 sample in 20 can be wrongly classified as negative (lower than STC). This specific t-value can be taken from a table for t-distribution and it is 1.729 for 20 samples set (19 degrees of freedom).

The false suspected rate is calculated based on the results obtained for 20 blank samples and 20 samples spiked at STC. The false suspected rate gives an estimation on how often the method will generate false suspect result. A 5% false suspected rate means that 1 sample in 20 can be wrongly classified as suspect. The lower the false suspected rate the better, as it means that fewer false suspect samples will require analysis by a confirmatory method. The t-value can be calculated from the following equation:

t-value = (cut-off − mean_blank_)/SD_blank_

where mean_blank_ is the mean response of the 20 blank samples. SD_blank_ is the standard deviation of blank samples. The probability of false suspect samples can be then taken from a table for t-distribution.

The aim of the validation was to demonstrate the fitness for purpose of the developed ELISAs as the screening methods for the detection of mycotoxins at the set STC level and higher in different commodities.

Additionally, limit of detection (LOD) was calculated as:

LOD = mean_blank_ + 3 × SD_blank_

#### 4.8.1. T-2/HT-2 ELISA Validation: Cut-Off and False Suspected Rate

For the T-2/HT-2 ELISA the STC was set to 12.5 μg/kg for cereals and 7.5 μg/kg for baby food. Validation was first performed using rye and cereal based baby porridge samples. Forty sub-samples of rye and cereal based baby porridge were weighed in and then half of them were spiked at STC. The STCs were chosen to allow for the sensitive detection of T-2/HT-2 below the indicative limits set by Commission Recommendation 2013/165/EU [4]. To prepare spiked samples, 50 μL of the appropriate dilution of T-2 in acetonitrile was added to the sub-samples and left to equilibrate for 30 min. Samples were analysed under intermediate precision conditions and the analysis was spread over 5 different days. Further, the method was tested for the detection of T-2 in cereals treated as a group and consisted of 20 samples of maize, barley, oat, malt and wheat (4 samples of each type). Similarly, baby food group comprised 20 baby food samples with different percentage of cereals and milk. Two sets of cereals and baby food were prepared, one was blank and the other was spiked at STC, which was 12.5 μg/kg for cereals and 7.5 μg/kg for baby food. Samples were analysed under intermediate precision conditions spread over 5 different days (4 samples of each set a day).

#### 4.8.2. T-2/HT-2 ELISA Validation: Recovery, Repeatability and Accuracy

Additional sets of samples were spiked at different concentrations with T-2 to determine recovery and repeatability of the developed method. The mean recovery and coefficient of variation (CV) were calculated for the results obtained by analysing 20 samples of rye spiked at 25, 50 and 100 μg/kg and 20 samples of baby porridge spiked at 7.5, 15, 30 μg/kg of T-2.

Accuracy was also determined by analysing 7 proficiency test and reference samples described in Section 4.6.1 and comparing the results obtained by ELISA to the assigned/reference values.

#### 4.8.3. OTA ELISA Validation: Cut-Off and False Suspected Rate

For the OTA ELISA, the STC was set at 2 μg/kg in wheat and corn; 0.4 μg/L in white wine, red wine and must; and 2.5 μg/kg in roasted, instant and green coffee and cocoa. The STC was chosen to allow for the sensitive detection of OTA below the maximum limits set by Commission Regulation (EC) No 1881/2006 and amendments [5]. During the validation sets of 20 blank samples (4 different samples, 5 replicates of each) and 20 samples (4 different samples, 5 replicates of each) spiked at STC were analysed under intermediate precision conditions, that is sample analysis was spread over 5 different days. Spiked samples were prepared by adding 50 μL of the appropriate dilution of OTA prepared in acetonitrile. The samples were left to equilibrate for 30 min before extraction.

#### 4.8.4. OTA ELISA: Recovery, Repeatability and Accuracy

Additional sets of samples were spiked at different concentrations with OTA to determine recovery and repeatability of the developed method. The mean recovery and coefficient of variation (CV) were calculated for the results obtained by analysing: 20 samples of corn and wheat spiked at 2 μg/kg, and 6 samples of corn and wheat spiked at 3 and 6 μg/kg; 20 samples of red wine, white wine and must spiked at 0.4 μg/L, and 6 samples of red wine, white wine and must spiked at 1, 2 and 4 μg/L; and 20 samples of roasted coffee, instant coffee, green coffee and cocoa spiked at 2.5 μg/kg, and 6 samples of roasted coffee, instant coffee, green coffee and cocoa spiked at 5 and 10 μg/kg.

Accuracy was also determined by analysing 14 proficiency test and reference samples described in Section 4.6.2 and comparing the results obtained by ELISA to the assigned/reference values.

## Figures and Tables

**Table 1:** Indicative levels for the sum of T-2 toxin and HT-2 toxin in food and feed [4].

Commodity	Indicative Levels (μg/kg)
Unprocessed cereals	100–1000
Cereal grains	50–100
Cereal products for human consumption	25–200
Cereal-based foods for infants and young children	15
Feed	250–2000

**Table 2:** Maximum limits for ochratoxin A in food [5].

Commodity	Maximum Limit (μg/kg)
Unprocessed cereals	5
Processed cereals	3
Dried vine fruit	10
Roasted coffee	5
Instant coffee	10
Wine and grape juice	2
Baby food and dietary food	0.5
Pepper, nutmeg, ginger, turmeric	15
Chili, cayenne, paprika	20
Liquorice	20

**Table 3:** LOD and results of the validation study for the T-2 toxin/HT-2 toxin ELISA in accordance with the guidelines included in Commission Regulation (EU) No 519/2014 [39].

	LOD ^b^ (μg/kg)	STC ^c^ (μg/kg)	Cut-Off (μg/kg)	False Suspected Rate (%)
Rye	8.9	12.5	10.6	<0.1
Cereals ^a^	12.0	12.5	9.5	2.5
Baby porridge	4.8	7.5	6.5	<0.1
Baby food	6.0	7.5	5.6	0.7

^a^ maize, barley, oat, wheat and malt; ^b^ limit of detection; ^c^ screening target concentration.

**Table 4:** Recovery and CV for the T-2 toxin/HT-2 toxin (T-2/HT-2) ELISA.

Matrix	Spiking Level (μg/kg) (*n* = 20)	Mean Concentration ± SD ^a^ (μg/kg)	Mean Recovery ± SD (%)	CV ^b^ (%)
Rye	12.5	14.2 ± 2.1	114 ± 17	14.7
	25	27.5 ± 3.2	110 ± 13	11.7
	50	52.6 ± 3.2	105 ± 6	6.1
Baby porridge	7.5	8.4 ± 1.1	112 ± 14	12.8
	15	16.6 ± 3.3	111 ± 22	20.2
	30	29.6 ± 2.0	99 ± 7	6.9

^a^ standard deviation; ^b^ coefficient of variation.

**Table 5:** Initial characterization of ochratoxin A (OTA) monoclonal antibodies by a competitive antigen-coated ELISA.

Fusion	Immunogen	Clone	Isotype	IC_50_^a^ OTA (ng/mL) (*n* = 3)
1	OTA-BSA	3A3	IgG1, κ	0.90
		9B10	IgG2a, κ	0.52
		17C7	IgG2b, κ	0.39
		18E2	IgG1, κ	0.13
2	OTA-KLH	16G3	IgG1, κ	6.51
		14A6	IgG2a, κ	9.08
		14F12	IgG1, κ	2.72
		16D4	IgG2a, κ	1.74
3	OTA-KLH	3A2	IgG2a, κ	2.99
		9C12	IgG2b, κ	0.07
		3G4	IgG2a, κ	1.07

^a^ 50% inhibitory concentration.

**Table 6:** LOD and results of the validation study for the ochratoxin A ELISA in accordance with the guidelines included in Commission Regulation (EU) No 519/2014 [39].

	LOD ^a^ (μg/kg)	STC ^b^ (μg/kg)	Cut-Off (μg/kg)	False Suspected Rate (%)
Wheat	1.7	2	1.8	0.1
Corn	1.4	2	1.9	<0.1
Red wine	0.3 ^c^	0.4 ^c^	0.3 ^c^	0.1
White wine	0.3 ^c^	0.4 ^c^	0.3 ^c^	1
Must	0.2	0.4	0.2	1
Roasted coffee	1.9	2.5	2.5	<0.1
Instant coffee	1.8	2.5	1.9	0.3
Green coffee	1.2	2.5	2.0	<0.1
Cocoa	1.7	2.5	1.7	0.4

^a^ limit of detection; ^b^ screening target concentration; ^c^ μg/L.

**Table 7:** Recovery and CV for the ochratoxin A (OTA) ELISA.

Matrix	Spiking Level (μg/kg)	Mean Concentration ± SD ^d^ (μg/kg)	Mean Recovery ± SD ^d^ (%)	CV ^e^ (%)
Wheat	2 ^a^	2.3 ± 0.3	115 ± 14	11.7
	3 ^b^	3.0 ± 0.2	99 ± 7	6.5
	6 ^b^	5.7 ± 0.2	96 ± 4	4.4
Corn	2 ^a^	2.1 ± 0.1	107 ± 7	6.8
	3 ^b^	3.1 ± 0.2	105 ± 5	4.9
	6 ^b^	5.7 ± 0.1	95 ± 1	1.5
Red wine	0.4 ^a,c^	0.4 ± <0.1 ^c^	97 ± 11	11.6
	1 ^b,c^	1.0 ± 0.2 ^c^	97 ± 15	15.7
	2 ^b,c^	1.9 ± 0.1 ^c^	94 ± 5	5.3
	4 ^b,c^	3.5 ± 0.4 ^c^	88 ± 11	12.1
White wine	0.4 ^a,c^	0.4 ± 0.1 ^c^	93 ± 15	15.7
	1 ^b,c^	1.0 ± 0.1 ^c^	104 ± 8	7.3
	2 ^b,c^	1.9 ± 0.1 ^c^	94 ± 8	7.9
	4 ^b,c^	3.8 ± 0.3 ^c^	94 ± 7	7.7
Must	0.4 ^a^	0.4 ± 0.1	89 ± 16	17.6
	1 ^b^	0.7 ± 0.1	68 ± 9	12.6
	2 ^b^	1.9 ± 0.2	94 ± 9	9.2
	4 ^b^	3.6 ± 0.3	90 ± 8	8.5
Roasted coffee	2.5 ^a^	2.8 ± 0.2	114 ± 9	7.5
	5 ^b^	5.6 ± 0.1	112 ± 3	2.4
	10 ^b^	10.3 ± 0.3	103 ± 3	2.5
Instant coffee	2.5 ^a^	2.5 ± 0.3	99 ± 14	14.2
	5 ^b^	5.3 ± 0.1	106 ± 3	2.4
	10 ^b^	9.6 ± 0.2	96 ± 2	1.7
Green coffee	2.5 ^a^	2.5 ± 0.3	101 ± 13	12.6
	5 ^b^	5.2 ± 0.1	104 ± 2	1.9
	10 ^b^	10.0 ± 0.3	101 ± 3	3.2
Cocoa	2.5 ^a^	2.30 ± 0.4	94 ± 16	17.2
	5 ^b^	3.8 ± 0.1	76 ± 3	3.7
	10 ^b^	7.9 ± 0.3	79 ± 3	3.6

^a^ *n* = 20; ^b^ *n* = 6; ^c^ μg/L; ^d^ standard deviation; ^e^ coefficient of variation.
